# Clinical and imaging characteristics of breast ductal carcinoma *in situ* with microinvasion

**DOI:** 10.1002/acm2.13122

**Published:** 2020-12-17

**Authors:** Sijia Han, Fang Qiu, Ye Han, Yongqing Xu, Jianqiao Yin, Fei Xing, Xiaobo Bian, Guijin He

**Affiliations:** ^1^ Department of Oncology Shengjing Hospital of China Medical University Shenyang 110004 China

**Keywords:** ductal carcinoma in situ, mammography, ultrasound

## Abstract

**Background:**

We analyzed the clinical and imaging characteristics of patients with breast ductal carcinoma *in situ* with microinvasion (DCISM) and breast ductal carcinoma *in situ* (DCIS).

**Methods:**

We analyzed the records of 40 patients diagnosed with DCISM and 61 patients with DCIS who were hospitalized at Shengjing Hospital (Shenyang, China) from January 2009 to June 2016. The size, hardness, and degree of calcification of tumors were determined by mammography and ultrasonography.

**Results:**

In all, 37 DCISM patients and 45 DCIS patients showed clinical palpable masses (92.5% vs 73.77%, *P* = 0.018). Mammography showed that the mean size of tumor was larger in DCISM patients than that of DCIS patients (3.13 ± 1.51 vs 2.68 ± 1.77, *P* = 0.030). Ultrasound examination revealed calcification shadows in the solid tumor mass in 17 DCISM cases and 11 DCIS patients (42.5 vs 18.03%, *P* = 0.007). Furthermore, estrogen receptor positivity and progesterone receptor positivity were more common in DCIS patients (32.5% vs 54.10%, *P* = 0.033; 22.5% vs 45.90%, *P* = 0.017), and the percentage of menopausal patients were higher in DCISM patients than that of DCIS patients (70.00% vs 47.54%, *P* = 0.026).

**Conclusion:**

Clinically palpable and calcified tumor masses on sonography are more commonly encountered in DCISM lesions.

## 1 INTRODUCTION

The incidence of breast cancer has been on the increase in recent decades. Breast ductal carcinoma *in situ* with microinvasion (DCISM) is defined as ductal carcinoma *in situ* (DCIS) with tumor cells infiltrating the basal membrane and the infiltration less than 1 mm in diameter.^1^ DCISM is also referred as T1mic by American Joint Committee on Cancer (AJCC). Compared with DCIS, DCISM has a higher risk of distant metastasis.^2^, ^3^, ^4^ More DCIS cases are diagnosed in clinical practice while DCISM is not common and its incidence is <1% of breast cancer.^5^, ^6^, ^7^ Previous studies mainly focused on the pathological features of DCISM. Meanwhile, the clinical and radiological characteristics of DCISM have not been clearly reported.^3^, ^5^, ^8^

Mammography and ultrasound have been used for diagnosing breast diseases. However, whether mammography and ultrasound could benefit the diagnosis of DCISM and DCIS is unknown.^9^, ^10^ Yoon et al. recently have shown that DCIS patients with suspected MRI features, negative progesterone receptor (PR), and high Ki‐67 levels are more likely to have invasion.^11^ The advantage of Breast MRI is more sensitive in detecting DCISM. However, MRI is more invasive than mammography and results in more false positives. Yao et al. studied 160 DCIS lesions and 58 DCISM lesions and found that DCISM was more likely to have microcalcifications and a high degree of vascularization than DCIS.^6^ Nonetheless, only sonography, which is less sensitive than mammography for the identification of calcifications, was used in this study. Mammography is well established and extensively used imaging method for the detection of DCIS. Wang et al. compared the sonographic and mammographic features of patients with DCIS and DCISM^12^ and showed that DCISM was associated with calcifications and vascularity on sonography or a lager distribution of calcifications on mammography. Accordingly, imaging features incorporating mammography (for larger area of calcification) and sonography (for calcification and vascularity) is the optimal way for identifying DCISM. In this study, we compared the radiological and clinical features of DCISM and DCIS, which may help to comprehensively understand DCISM in clinical diagnosis.

## 2 MATERIALS AND METHODS

### 2.1 Patients

**Fig. 1:**
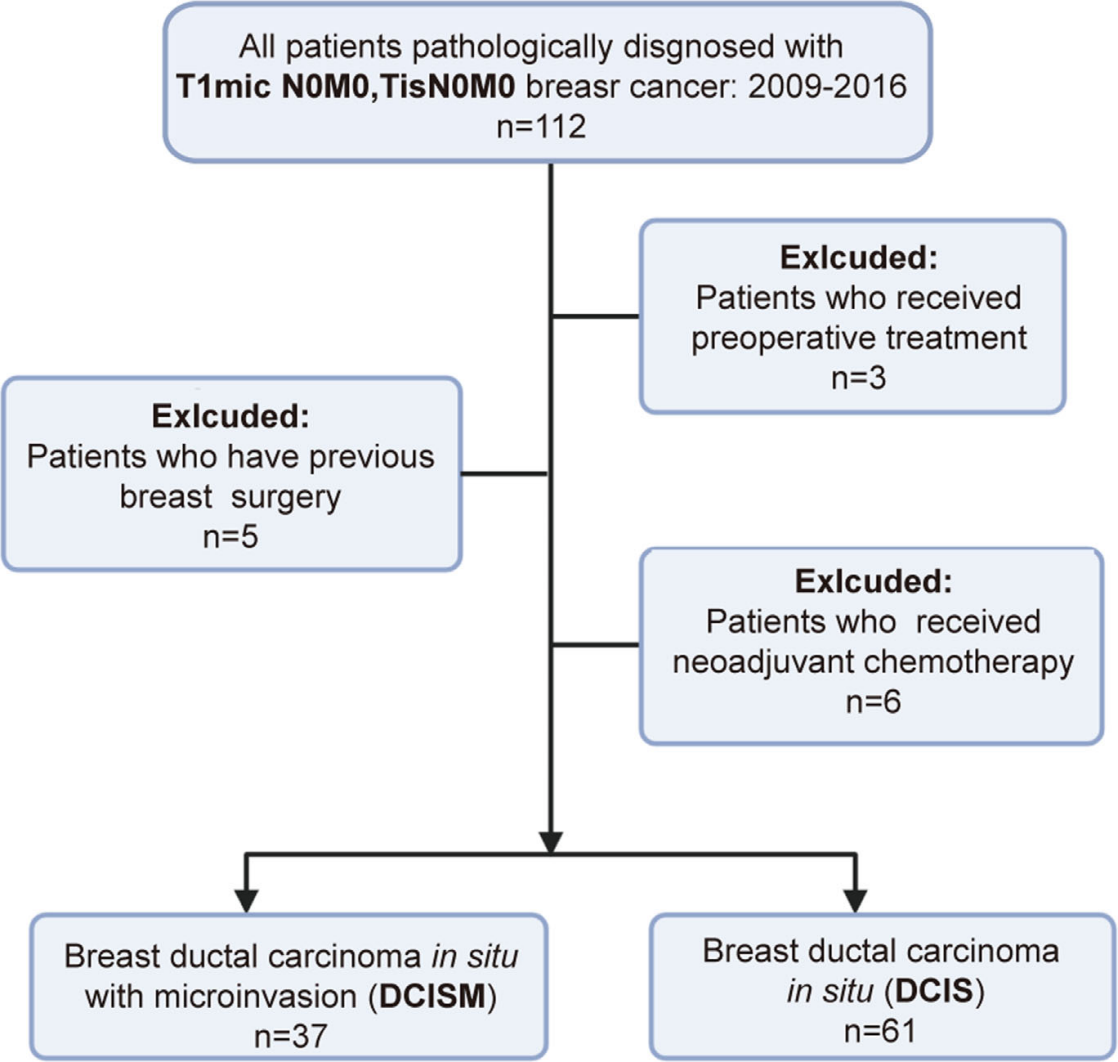
Patient inclusion–exclusion criteria.

A retrospective study was designed for the enrollment of patients. From January 2009 to June 2016, hospitalized patients in Oncology Department of Shengjing Hospital (Shengyang, China), who underwent surgical resection and were pathologically diagnosed with T1mic N0M0 breast cancer were included in the study. Patients who received preoperative treatment were excluded from the study. A total of 101 patients (40 patients with breast DCISM and 61 patients with DCIS) were enrolled (see the patient inclusion–exclusion criteria in Fig. 1). Demographic data and clinical and radiological characteristics including pathology were collected and analyzed.

### 2.2 Radiological and ultrasound examinations

The size of breast neoplasm and its calcification degree were detected with mammography, and the distort and disorder of tumor structure was detected with ultrasound. If the lesion was unclear under mammography, ultrasound was used to measure the size of the lesion. The maximal diameter of neoplasm or calcification lesion was determined with ultrasound. Ultrasonograms and mammograms were reviewed retrospectively by two breast imaging radiologists with more than 5 yr of experience in breast ultrasonography who were blind to the pathologic data of the patients. Difference between the two radiologists was resolved by involving a third radiologist to reach a consensus.

Sonographic examinations were performed using an Aplio400 (Toshiba Medical Systems, Japan). The probe frequency was 4–15 MHz. The SMI had color mode (cSMII) or monochrome mode (mSMI) and CDFI was used for observing blood flow in the tumor. Two vertical images of each tumor were obtained. Ultrasonograms were reviewed retrospectively by two breast imaging radiologists with more than 5 yr of experience in breast ultrasonography who were blind to the pathologic data of the patients. Difference between the two radiologists was resolved by involving a third radiologist to reach a consensus. The sonographic findings including shape, orientation, margin, echo pattern, and posterior features were described using the American College of Radiology Breast Imaging Reporting and Data System (BI‐RADS) lexicon.^13^ Mammography was done using FDR MS‐3500 with two standard imaging planes (mediolateral oblique and craniocaudal) and imaging analysis was done by two experienced breast imaging radiologists and types of lesions including mass and calcification, asymmetry, density, and microcalcification and their distribution were analyzed using the BI‐RADS.

### 2.3 Statistical analysis

Continuous variables were expressed as mean±standard deviation. Normally distributed data were analyzed with Student's *t* test and with non‐normally distributed data were analyzed with Wilcoxon two sample tests. Categorical data were expressed as frequency (%) and were analyzed using Chi‐square test or the Fisher exact test. Statistical analysis was carried out by SPSS 15.0 (SPSS Inc., Chicago, IL, USA). P < 0.05 (two‐sided) indicated statistically significant difference.

## 3 RESULTS

### 3.1 Clinical characteristics

The mean age of the DCISM patients was 51.45 ± 11.64 yr, 28 (70%) of them were menopausal and 2 did not bear children. One (2.50%) patient had a family history of breast cancer. In the DCISM group, 37 (92.50%) patients had a palpable mass and the mean size of the mass was 3.13 ± 1.51 cm. One patient had bloody nipple discharge. The mean age of DCIS patients was 51.87 ± 10.17 yr, 29 (47.54%) of them were menopausal and 1 (1.64%) patient had a family history of breast cancer. In all, 45 (73.77%) patients in the DCIS group had a palpable mass and no patients had bloody nipple discharge (Table 1). More patients with DCISM had menopause (P = 0.026) and a palpable mass (P = 0.030) with a larger size (P = 0.018) while more DCIS patients had estrogen receptor (ER) positivity (P = 0.033) and PR positivity (P = 0.017).

**Table 1:** Demographic and clinical characteristics.

	DCISM, n = 40	DCIS, n = 61	P	
Age, mean ± SD, years old	51.45 ± 11.64	51.87 ± 10.17	0.878	
Menopause, n (%)	28 (70.00)	29 (47.54)	**0.026**	
Breast cancer history of family, n (%)	1 (2.50)	1 (1.64)	1.000	
Size (mean ± SD, cm)	3.13 ± 1.51	2.68 ± 1.77	**0.030**	
Palpable mass	37 (92.50)	45 (73.77)	**0.018**	
Nipple discharge	1 (2.50)	0 (0.00)	0.396	
Lymph node metastasis	1 (2.50)	1 (1.64)	1.000	
ER positivity	13 (32.50)	33 (54.10)	**0.033**	
PR positivity	9 (22.50)	28 (45.90)	**0.017**	
HER positivity	18 (45.00)	18 (29.51)	0.112	

Bold is indicating *P* value less than 0.05, which is considered as significant difference.

DCISM: ductal carcinoma *in situ* with microinvasion, DCIS: ductal carcinoma *in situ*.

### 3.2 Radiological findings

In the DCISM group, tumor microcalcification was characterized in 28 patients, 15 of whom had masses, 6 had irregular structure, and 7 had only microcalcification. Mass without other abnormality was found in seven patients. Normal mammography was observed in two patients who had <1 cm mass without calcification in both breasts. Ultrasound data of 40 patients with DCISM were collected and masses were detected in 30 patients including 17 patients with calcification. Pure calcification was found in two patients and irregular structure and calcification were observed in five patients. No statistical difference was found in the diagnostic rate by ultrasound and mammography (Table 2).

**Table 2:** Radiological characteristics.

	DCISM, n = 40 n (%)	DCIS, n = 61 n (%)	P
**Ultrasound abnormality**	**39 (97.50)**	**51 (83.61)**	**0.062**
Solid mass with calcification	17 (42.50)	11 (18.03)	0.007*
Pure solid mass shadow	13 (32.50)	28 (45.90)	0.180
Pure calcification	2 (5.00)	5 (8.20)	0.827
Irregular structure with calcification	5 (12.50)	3 (4.92)	0.316
Irregular structure	2 (5.00)	4 (6.56)	1.000
**X‐ray abnormality**	**38 (95.00)**	**57 (93.44)**	**1.000**
Pure microcalcification	7 (17.50)	20 (32.79)	0.090
Pure mass shadow	7 (17.50)	16 (26.23)	0.306
Microcalcification with mass	15 (37.50)	13 (21.31)	0.075
Irregular structure with microcalcification	6 (15.00)	6 (9.84)	0.638
Irregular structure	3 (7.50)	2 (3.28)	0.626

* *P* < 0.05.

**Fig. 2:**
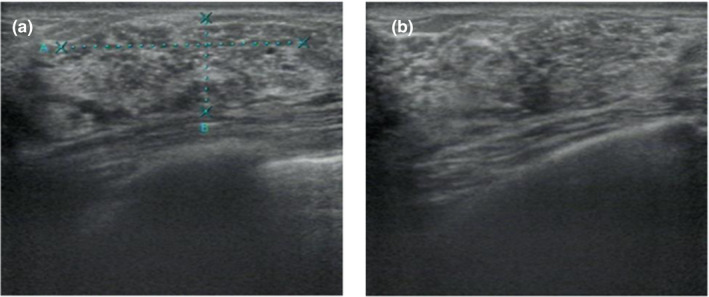
(a), A 45‐yr‐old ductal carcinoma in situ female patient. Mammography reveals multiple irregular microcalcifications in segmental distributions. (b), Breast ultrasound of the same patient shows multiple punctate hyperechoic flocculent foci.

In 61 DCIS patients, mammography detected pure microcalcification in 20 patients, pure mass shadow in 16 patients, microcalcification with mass in 13 patients, irregular structure with microcalcification in 6 patients and irregular structure in 2 patients (Fig. 2 and Table 2). Ultrasound showed solid mass with calcification in 11 patients, pure solid mass shadow in 28 patients, pure calcification in 5 patients, irregular structure with calcification in 3 patients, and irregular structure in 4 patients (Fig. 2). Solid mass with calcification on ultrasound was more common in DCISM (*P* = 0.007) while microcalcification on mammography was more common in DCISM (*P* = 0.075).

### 3.3 DCISM could be clinically differentiated from DCIS

**Fig. 3:**
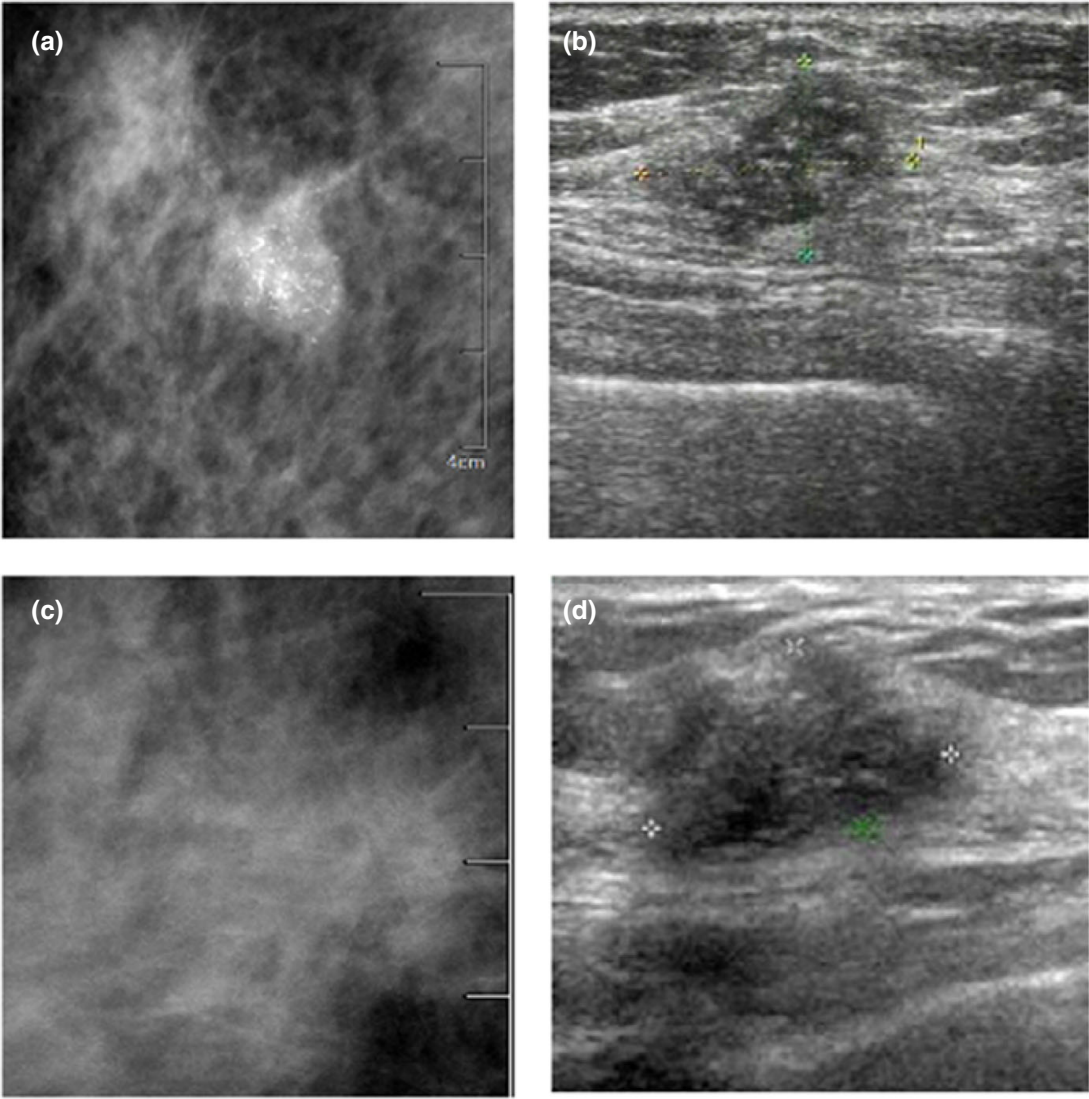
(a) A 57‐yr‐old ductal carcinoma in situ with microinvasion (DCISM) female patient. A1, mammography detects the presence of round high‐density node with a clear border and multiple calcifications. A2, Ultrasound shows a hypoechoic mass with an unclear border and multiple hyperechoic spots.(b), A 45‐yr‐old DCISM female patient. B1, mammography reveals a solid mass with an unclear border and irregular morphology. B2, Ultrasound shows the hypoechoic irregular mass with an unclear border.

The clinical and radiological findings were compared between DCISM and DCIS. The percentage of menopausal patients were higher in the DCISM group than in the DCIS group (70.00% vs 47.54%, *P* = 0.026). The mass size of the DCISM group was larger than that of the DCIS group (3.13 ± 1.51 vs 2.68 ± 1.77, *P* = 0.030). More palpable masses were detected in DCISM patients (92.5% vs 73.77%, *P* = 0.018). ER positivity and PR positivity were more common in DCIS patients (32.5% vs 54.10%, *P* = 0.033; 22.5% vs 45.90%, *P* = 0.017). DCISM often manifested as a calcified solid mass on ultrasound (42.5% vs 18.03%, *P* = 0.007), and a microcalcified mass on mammography (37.50% vs 21.31%, *P* = 0.075) (Fig. 3).

## 4 DISCUSSION

DCIS has been uncommon until breast mammography has been widely applied in clinical practice. The percentage of newly diagnosed breast DCIS has been on the rise recently, but DCISM is still rare.^10^, ^14^, ^15^ It was reported that <1% of breast cancer was confirmed as DCISM, which is similar to the percentage observed in our patient cohort. DCISM is defined as infiltration of breast tumor cells into the basal membrane and surrounding tissues in a diameter less than 1 mm by AJCC. If there are more than one microinvasion lesions, the maximal diameter of the lesion is used for classification rather than the sum of diameters of all the lesions.^1^ We hypothesized that DCISM was a transition from DCIS to early infiltrative tumor. Besides, as the tumor grows fast, once the tumor cells infiltrate the basal membrane, the infiltration lesion will exceed 1 mm, which may be the main reason why DCISM is rare.

At present, DCISM of the breast is considered T1mic. There is still a controversy on whether the pathology of DCISM is different from that of DCIS, indicating that making an accurate pathological diagnosis is of vital importance. DCISM has a risk of metastasis, and it is estimated that the incidence of lymph node metastasis in DCISM is 0–14%.^1^, ^14^, ^16^ The therapeutic regimen for stage 0 and 1 is different, so the clinical characteristics and treatments for DCISM need to be further investigated.

Based on our results, 92.5% of DCISM patients had palpable mass, which is higher than that of DCIS (*P* < 0.05). Yang et al.^17^ reported that 93% (26/28) patients had palpable mass. de Mascarel et al.^18^ showed that DCISM was more prone to have palpable mass than DCIS. Intra et al.^19^ also reached a similar conclusion that a majority of DCISM had specific clinical manifestations. We found that the mass was detected by mammography or ultrasound in over 90% of DCISM patients, which is different from that of DCIS patients (*P* < 0.05). Microcalcified mass was the most common characteristic of DCISM compared with DCIS. The diagnostic rate of ultrasound and mammography for DCISM was comparable. Ultrasound has an advantage of detecting <1 cm mass without calcification. Mass with microcalcification is common in DCISM while pure calcification is common in DCIS, which could be used for differentiating DCISM from DCIS. Mass with calcification on ultrasound was significantly different between DCISM and DCIS, but no significant difference was found in the mammographic characteristics, which may be explained by the fact that the presence of mass is not obvious in some cases with multiple glands or solid gland.

There are still limitations in our study. There is no method of accurately measuring the size of the mass. We estimated the lesion by mammography or ultrasound, which may result in overestimation or underestimation. Taken together, a large proportion of DCISM patients have palpable mass and abnormal mammography or ultrasound findings. The clinical and radiological features of DCIS are different from those of DCISM. The presence of mass with calcification on ultrasound and palpable mass are common in DCISM and may predict the occurrence of microinvasion, which could help the clinicians and pathologists in diagnosing DCISM.

## 5 CONCLUSION

In this study, we demonstrated that clinically palpable mass and calcified mass on ultrasound are commonly encountered in DCISM lesions.

## AUTHORS' CONTRIBUTION

GH, SH, and FQ contributed to the study design. All authors collected the data and performed the data analysis. All authors prepared the manuscript. GH, SH, FQ, and YH amended the manuscript critically.

## CONFLICT OF INTEREST

All the authors declare that they have no conflict of interest.

## ETHICS APPROVAL AND CONSENT TO PARTICIPATE

Ethical approval was given by the Ethics Committee of Shengjing Hospital of China Medical University. All patients gave their written information consent.

## Data Availability

The datasets used and/or analyzed during the current study are available from the corresponding author on reasonable request.
